# Kidney Complications of COVID-19: A Systematic Review and Meta-Analysis

**DOI:** 10.34172/jrhs.2021.39

**Published:** 2021-01-12

**Authors:** Naser Nasiri, Shoboo Rahmati, Abbas Etminan, Hamid Sharifi, Azam Bazrafshan, Mohammad Karamouzian, Ali Sharifi

**Affiliations:** ^1^Student Research Committee, Kerman University of Medical Sciences, Kerman, Iran; ^2^Department of Internal Medicine, School of Medicine, Endocrinology and Metabolism Research Center, Afzalipour Hospital, Kerman University of Medical Sciences, Kerman, Iran; ^3^HIV/STI Surveillance Research Center, and WHO Collaborating Center for HIV Surveillance, Institute for Futures Studies in Health, Kerman University of Medical Sciences, Kerman, Iran; ^4^School of Population and Public Health, Faculty of Medicine, University of British Columbia, Vancouver, BC, Canada; ^5^Department of Ophthalmology, Shafa Hospital, Afzalipour School of Medicine, Kerman University of Medical Sciences, Kerman, Iran

**Keywords:** Prevalence, Acute kidney injury, COVID-19, Blood urea nitrogen, Creatinine

## Abstract

**Background:** Some patients with coronavirus disease 2019 (COVID-19) have been reported to have developed mild to severe kidney injuries. The current systematic review and meta-analysis was carried out to estimate the prevalence and incidence of acute kidney injury (AKI) among COVID-19 patients.

**Study design:** A systematic review and meta-analysis

**Methods:** PubMed, Embase, Scopus, Web of Science, and MedRxiv databases were searched from December 1, 2019, up to July 27, 2020. Two independent co-authors completed the screening process, data extraction, and quality assessment of the retrieved records. Random-effects meta-analyses were used to determine the pooled prevalence and 95% confidence interval (CI) of AKI among COVID-19 patients.

**Results:** Out of 2,332 unique identified records, 51 studies were included in the review. Overall, the studies were carried out on 25,600 patients. A total of 6,505 patients (in 18 cross-sectional studies) were included to estimate the pooled prevalence of AKI, and 18,934 patients (in 27 cohort studies) were included to determine the pooled incidence of AKI. The pooled prevalence of AKI was estimated as 10.08% (95% CI: 4.59, 17.32; I ^2^=98.56%; *P*<0.001). Furthermore, the pooled incidence of AKI was 12.78% (95% CI: 7.38, 19.36; I ^2^=99.27%; *P*<0.001). The mean (95% CI) values of serum creatinine (SCr), blood urea nitrogen (BUN), potassium, and sodium were 76.10 (69.36, 82.84), 4.60 (4.04, 5.30), 3.94 (3.78, 4.11), and 139.30 (138.26, 140.36) mmol/L, respectively.

**Conclusions:** The AKI is a considerable complication among COVID-19 patients and should be screened for on clinical examinations. The BUN, SCr, potassium, and sodium levels were within the normal ranges.

## Introduction

Severe acute respiratory syndrome coronavirus 2 (SARS-CoV-2) rapidly spread around the globe^1^, and coronavirus disease 2019 (COVID-19) was declared a pandemic by the World Health Organization in March 2020 ^2^. By October 11, 2020, more than 37 million individuals had been infected with COVID-19, and 1,078,715 cases had died due to COVID-19 ^3^. The SARS-CoV-2 enters the host cells and binds to angiotensin-converting enzyme 2 (ACE2) receptors^1^. Most COVID-19 patients have multiple signs or symptoms; however, commonly reported signs and symptoms are fever, fatigue, dry cough, and muscular pain ^4^. COVID-19 is more common among individuals with multimorbidities, such as hypertension, diabetes, cardiovascular, and respiratory diseases ^5^.

Different internal organs, such as heart, liver, and kidney, can be damaged in COVID-19 patients ^6^. The results of studies showed that ACE2 is the central receptor to enter pathogens in human cells ^7,8^. The ACE2 is expressed in kidneys ^9^ and exists in proximal tubule cells ^8^. The SARS-CoV-2 can particularly affect kidneys and lead to acute kidney injury (AKI) ^9^. Several studies have reported AKI and increased blood urea nitrogen (BUN) and serum creatinine (SCr) in COVID-19 patients ^6,10,11^. For example, Yang et al. reported that 13.7% and 9.6% of COVID-19 patients had elevated BUN and SCr levels, respectively ^10^. Moreover, AKI has also been shown to increase the probability of mortality among COVID-19 patients, and different studies have reported varying prevalence of AKI ^5,11,12^. The present systematic review aimed to summarize the prevalence and incidence of AKI and mean laboratory tests in COVID-19 patients.

## Methods

According to the systematic review and meta-analysis (PRISMA) checklist (see supplementary file S1 for PRISMA checklist), ^13^ several databases, including PubMed, Scopus, Embase, Web of Science, and MedRxiv, were searched from December 1, 2019, up to July 27, 2020. The search terms were combined using appropriate Boolean operators and adjusted for different databases. The search concepts included subject heading terms/keywords for kidney complications (e.g., "Injury", "Impairment", "Nephro", "Kidney", or "Renal") and SARS-CoV-2 (e.g., "COVID-19" or "Coronavirus"). Supplementary file S2 shows a sample search strategy.

###  Inclusion criteria and study selection

 Studies reporting on the kidney complications of COVID-19 with any design, including case reports, case series, cohorts, and cross-sectional studies, were eligible for this systematic review. However, only cross-sectional and cohort studies were considered for meta-analysis. Non-original studies, such as editorials, letters to the editor, commentaries, and reviews, were excluded from the study. Studies obtained through the electronic database searching were transferred to EndNote software version X8. Two independent co-authors (SHR and AB) completed the title, abstract, and full-text screening process. Any disagreement on the process of selected studies was resolved by discussion with the senior co-author and a nephrologist co-author (ASH and AE).

###  Data extraction 

 Data were independently extracted from the eligible studies by two co-authors (NN and SHR). The items considered in data extraction included study characteristics (e.g., first author, publication date, study design, study location, and sample size) and participant characteristics (e.g., age, sex, kidney complications, and laboratory test results).

###  Quality assessment of the evidence 

The Joanna Briggs Institute critical appraisal tool was used to assess the quality of the included studies ^14^. This tool evaluates quality with differential items, including 8 items for case report studies, 10 items for case series, 9 items for cross-sectional studies, and 11 items for cohort studies.

###  Statistical analysis

Descriptive statistics (i.e., mean, median, and standard deviation [SD] for continuous variables and frequency and percentage for categorical variables) were used in this study. Weighted means were utilized to estimate the pooled mean and 95% confidence interval (CI) of laboratory tests. The *Metaprop* command in Stata software was used to determine the pooled prevalence and incidence of AKI and associated 95% CI of kidney complications. Random-effects meta-analysis was used in the study. The heterogeneity among the included studies was assessed using I^2^ and Q-statistic. A value of ≥ 50% of I^2^ and p-value of < 0.1 for the Q-statistic were regarded as considerable heterogeneity. Then, a meta-regression was fitted to assess the potential sources of heterogeneity. For fitting the meta-regression, several variables, namely diagnostic method of COVID-19 (i.e., polymerase chain reaction [PCR] vs. computed tomography [CT] scan and clinical signs), quality of studies (i.e., a quality score of ≥ 4 vs. quality score of < 4), number of study centers (i.e., multi-center vs. single-center), and recruited sample size (i.e., a sample size of > 500 vs. sample size of ≤ 500), were included. Stata software (version 14.2) was used for all statistical analyses. In addition, all comparisons were two-tailed with a threshold p-value of ≤ 0.05 for statistical significance.

## Results

**Figure 1:**
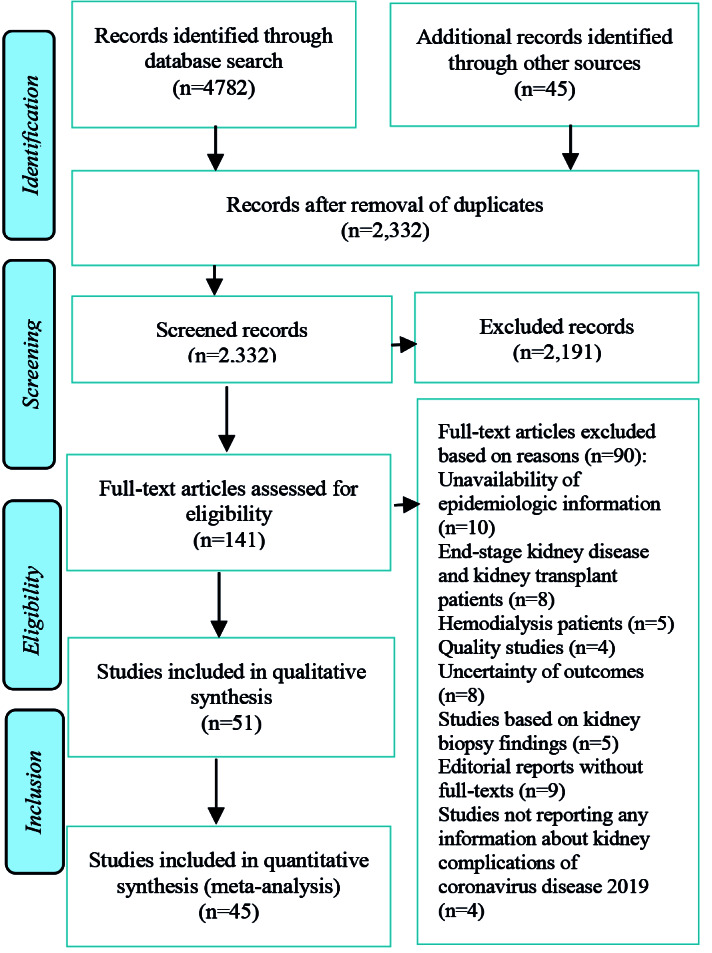
Flowchart of studies included in the systematic review of coronavirus disease 2019 kidney injury

Out of the 2,332 retrieved unique records, 51 studies were included in the present systematic review ^4,5,15-63^ (Figure 1). Four studies were case reports ^60-63^, and two studies were case series ^28,29^. Furthermore, 27 studies were cohort ^4,5,16,17,19,21,24,27,31,33-35,37-39,46,47,49-56,58,59^, and 18 studies were cross-sectional ^15,18,20,22,23,25,26,30,32,36,40-45,48,57^. Moreover, 47 and 4 studies reported aggregate-level ^4,5,15-59^ and individual-level^60-63^ information about kidney complications, respectively. Supplementary file S3 shows further details (e.g., population data, location, sex, age, and chronic disease) about the studies included in the present systematic review.

Overall, the studies were carried out on 25,600 patients with COVID-19. The number of patients in different studies was within the range of 1-5,449 patients. Most cases were male (12,294 out of 22,008; 56.0%), and the age range of the participants was within 1 month to 96 years. The SARS-CoV-2 diagnosis was confirmed in 16,598 patients (64.8%) with PCR and 9,002 subjects (35.2%) using clinical signs and CT scans. The most diagnosed comorbidities in patients were hypertension (9,527 out of 22,360; 42.6%), diabetes (5,241 out of 22,360; 23.4%), cardiovascular diseases (2,390 out of 22,360; 10.7%), respiratory diseases (1,886 out of 22,360; 8.4%), and cancer (1,234 out of 22,360; 5.5%). Table 1 tabulates the demographic and clinical characteristics of the patients included in the studies.

**Table 1:** Demographic and clinical characteristics of coronavirus disease 2019 patients included in the reviewed studies

**Characteristics**	**n**	**%**
Diagnostic approach (n=25,600)		
Only clinical signs and computed tomography scans	9,002	35.2
Confirmed polymerase chain reaction	16,598	64.8
Sex (n=22,008)		
Male	12,294	56.0
Female	9,714	44.0
Comorbidity with COVID-19 (n=22,360)		
Hypertension	9,527	42.6
Diabetes	5,241	23.4
Cardiovascular diseases	2,390	10.7
Respiratory system diseases	1,886	8.4
Cancer	1,234	5.5
Renal diseases	1,131	5.1
Liver diseases	436	2.0
Cerebrovascular diseases	388	1.7
Autoimmune diseases	127	0.6

COVID-19: Coronavirus disease 2019

###  Quality assessment of included studies

 The overall quality of the studies was low. The scores of the case report, case series, cross-sectional, and cohort studies were within the ranges of 2-5 (out of 8 possible points), 2-3 (out of 10 possible points), 1-6 (out of 9 possible points), and 3-7 (out of 11 possible points), respectively. Supplementary file S4 shows further details in this regard.

###  Pooled prevalence of AKI

**Figure 2:**
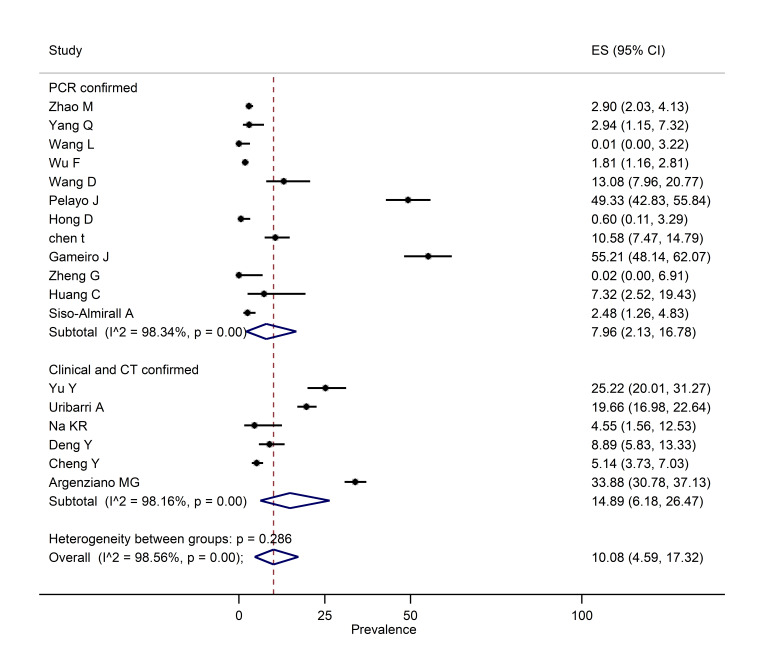
Pooled prevalence of kidney injury among patients with coronavirus disease 2019

The pooled prevalence of AKI was estimated based on the data obtained from 18 cross-sectional studies carried out on 6,505 patients (Figure 2). The pooled prevalence of AKI was estimated as 10.08% (I^2^=98.56; 95% CI: 4.59, 17.32; *P*<0.0001).

###  Pooled incidence of AKI

**Figure 3:**
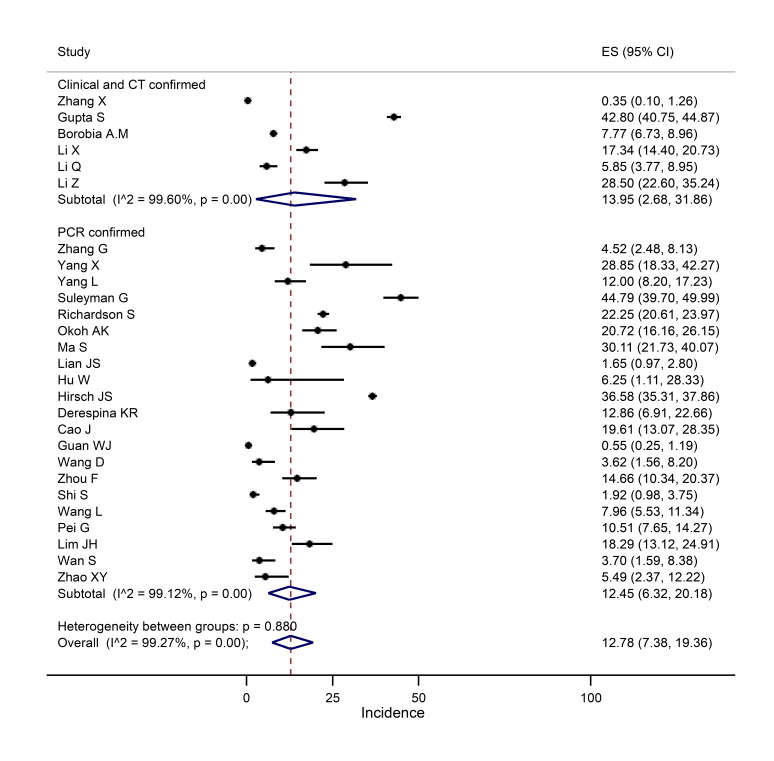
Pooled incidence of kidney injury among patients with coronavirus disease 2019

The pooled incidence of AKI was estimated based on the data obtained from 27 cohort studies conducted on 18,934 patients (Figure 3). The pooled incidence of AKI was estimated as 12.78% (I^2^=99.27; 95% CI: 7.38, 19.36; *P*<0.0001).

###  Biochemical tests

The mean (95% CI) values of SCr, BUN, potassium, and sodium among patients diagnosed with COVID-19 were estimated as 76.10 (69.36, 82.84), 4.60 (4.04, 5.30), 3.94 (3.78, 4.11), and 139.30 (138.26, 140.36) mmol/L, respectively (Table 2).

###  Meta-regression

The results of meta-regression did not reveal the significant sources of heterogeneity among the studies included in the present meta-analysis (Table 3).

## Discussion

This systematic review and meta-analysis included 51 studies and 25,600 COVID-19 patients. Based on the included evidence, it was estimated that the prevalence and incidence of AKI were about 10% and 12%, respectively. These findings are comparable with previous results varying greatly across different studies. For example, in a study carried out by Pei et al. in China, the incidence of AKI was reported as 4.7% (22 out of 467) ^54^. In addition, Cao et al. estimated the incidence of AKI as 19.6% (n=22) among 102 Chinese patients ^4^. In the United States, Hirsch et al. studied 5,449 COVID-19 patients and estimated the incidence of AKI as 36.6% (n=1,993) ^37^. Moreover, previous systematic reviews have calculated the prevalence of AKI among COVID-19 patients within the range of 3-17% ^64,65^.

The AKI has also been reported in previous coronavirus pandemics. For example, Cha et al. reported the prevalence rates of AKI in Middle East Respiratory Syndrome coronavirus and severe acute respiratory syndrome patients to be 26.7% (8 out of 30) ^66^ and 6.7% (36 out of 536) ^67^, respectively. Kidneys have an essential role in drug metabolism. In addition, damages to kidneys and accumulation of drugs could increase toxin accumulation in patients ^68,69^. Kidney injuries increase the severity of the disease among COVID-19 patients and need for mechanical ventilation ^11,37^. In a recent meta-analysis, the pooled odds ratio for mortality among COVID-19 patients with AKI was reported as 13.3 ^11^. Paying further attention to kidney injuries in the clinical assessment of patients could help decrease COVID-19-related morbidities and mortalities.

**Table 2:** Laboratory tests of coronavirus disease 2019 patients included in the reviewed studies

**Test**	**Unit**	**Normal range**	**n**	**Mean (95% CI)**	**Range**	**N o. of studies**
Serum creatinine	μmol/L	70.72-114.92	12,887	76.1 (69.36, 82.84)	60-117.6	29
Blood urea nitrogen	μmol/L	3.1-8.0	4,507	4.6 (4.04, 5.30)	3.5-7.34	15
Serum potassium	μmol/L	3.5-5.3	3,541	3.94 (3.78, 4.11)	3.7-4.2	9
Serum sodium	μmol/L	137-147	5,162	139.3 (138.26, 140.36)	137-141.2	11
Creatine kinase	U/L	50-310	6,369	87.5 (59.13, 115.93)	3.85-164	16
Procalcitonin	ng/mL	0.0-0.5	3,472	0.09 (0.04, 0.15)	0.05-0.1	6
C-reactive protein	mg/L	0-8	6,910	84.2 (24.97, 143.50)	3.4-158	16

**Table 3:** Meta-regression of the effect of the factors on the acute kidney injury of coronavirus disease 2019 patients

**Variable**	**Prevalence**			**Incidence**		
	**Coefficient**	**95% CI**	***P*** **-value**	**Coefficient**	**95% CI**	***P*** **-value**
Quality score of the included papers (i.e., ≥4 vs. <4)	0.01	-0.17, 0.19	0.906	0.05	-0.07, 0.18	0.406
Sample size (i.e., ≤500 vs. >500)	0.03	-0.21, 0.28	0.757	-0.04	-0.20, 0.11	0.581
Diagnostic method (i.e., polymerase chain reaction vs. computed tomography scans and clinical signs)	- 0.08	-0.29, 0.13	0.433	-0.04	-0.19, 0.10	0.541
Type of study (i.e., multi-center vs. single-center)	-0.12	-0.35, 0.10	0.261	0.04	-0.08, 0.17	0.496

The weighted mean values of SCr and BUN were 76.1 (range: 60-117.6) and 4.6 (range: 3.5-7.34) mmol/L, respectively. The normal ranges of SCr and BUN are 70.72-114.92 and 3.1-8 mmol/L, respectively. Laboratory tests could be used to diagnose AKI at the early stages of the disease. When used at an appropriate time, laboratory tests can can help reach earlier diagnosis of kidney injuries kidney injuries ^11,70^. The BUN and SCr are crucial laboratory tests for the diagnosis of AKI ^11,70^ and may increase in COVID-19 patients ^10^. For example, Zhao et al. showed increased levels of SCr in 11.6% (116 out of 1,000) of COVID-19 patients ^15^. Increased BUN and SCr are indeed observed in severe COVID-19 patients in another study ^41^. Chen et al. demonstrated BUN levels in deceased patients to be higher than the normal range ^42^. However, the weighted mean values of BUN and SCr were within the normal ranges in the included studies. This could be due to the fact that the included studies calculated the weighted mean of BUN and SCr for all COVID-19 patients and not separately for those with AKI. Therefore, the assessment of BUN and SCr in COVID-19 patients with AKI would be informative.

The weighted mean values of potassium and sodium were 3.94 (range: 3.7-4.2) and 139.3 (range: 137-141.2) mmol/L, respectively. The normal ranges of potassium and sodium are 3.5-5.3 and 137-147 mmol/L, respectively. The assessment of the baseline levels of electrolytes can help evaluate the risk of mortality among COVID-19 patients ^71^. Sodium may also help predict progression to severe disease in COVID-19 patients ^72^. Sodium and potassium levels could be lower in severe COVID-19 cases ^73^. For example, Tezcan et al. reported that low baseline sodium was related to higher mortality in COVID-19 patients ^71^. In addition, hypernatremia can be a manifestation of COVID-19 and adverse outcomes in COVID-19 cases ^74^.

 Although sodium and potassium levels are important in COVID-19 patients, the current study showed sodium and potassium levels to be within the normal ranges. This could be due to the fact that the studies measured sodium and potassium only once during the course of the study. Therefore, it is suggested to carry out further studies to measure sodium and potassium levels in the initial admission of patients and continue monitoring the patients’ sodium and potassium levels throughout their hospital admission.

 The current study had several limitations. Firstly, the quality of studies included in the meta-analysis was low. Secondly, most studies had a low sample size. Thirdly, the variations between studies could lead to different measurements. Fourthly, the diagnostic methods were the use of PCR in some studies and clinical signs and CT scans in other studies that can be taken into account when interpreting the findings. Nonetheless, the findings of the present study are informative for the enhancement of the identification of AKI among COVID-19 patients.

## Conclusions

 The prevalence and incidence of AKI seem to be considerable among COVID-19 patients. The BUN, SCr, sodium, and potassium were within the normal ranges. The assessment and monitoring of COVID-19 patients for renal complications and comorbidities can help improve care and health outcomes among COVID-19 patients.

## Acknowledgements

 MK is a member of the COVID-19 Impact Committee of Pierre Elliott Trudeau Foundation and is supported by the Pierre Elliott Trudeau Foundation Doctoral Scholarship.

## Conflict of interest

 The authors declare that there is no conflict of interest.

## Funding

 This review received no external funding or other supports.

## Authors ʼ contributions

 All the authors contributed to conceptualization, study design, and data analysis. Screening was completed by SHR and AB. ASH and AE supervised the screening process. Data extraction was carried out by NN and SHR. Data analysis was performed by HSH and MK. NN, HSH, MK, and ASH wrote the first draft of the manuscript. The manuscript was read and approved by all the authors.

## Highlights

- Acute kidney injury is a prevalent complication among coronavirus disease 2019 (COVID-19) patients.
- Monitoring COVID-19 patients for renal injuries could help improve their health outcomes.
- Overall, blood urea nitrogen, serum creatinine, sodium, and potassium were within the normal ranges among the included patients in the review.
